# Army Veterinary Service

**Published:** 1900-09

**Authors:** 


### ARMY VETERINARY SERVICE.

New Jersey is going to be felt as a powerful influence in army legislation at Washington the coming session of Congress. The President of the Veterinary Medical Association of New Jersey has appointed a member of the profession from each congressional district to form a central committee of eight, with that active veterinarian of the Essex Troop, Dr. T. Earle Budd, as chairman. This committee will be ably assisted by some three to five veterinarians in each representative district, and there will be a demand for professional recognition with rank that will be backed by so powerful a sentiment that it will not be denied. The following are the committees:

*At Large:* Sixth congressional district, Dr. T. Earle Budd, chairman, 90 Park St., Orange; first congressional district, Dr. John O. George, Camden; second congressional district, Dr. L. P. Hurley, Hopewell; third congressional district, Dr. E. L. Loblein, New Brunswick; fourth congressional district, Dr. M. M. Stage, Dover; fifth congressional district, Dr. Wm. Herbert Lowe, 188–190 Ellison St., Paterson; seventh congressional district, Dr. T. E. Smith, 309 Barrow St., Jersey City; eighth congressional district, Dr. W. F. Harrison, Bloomfield.

*First Congressional District:* Camden, Cape May, Cumberland, Gloucester, and Salem Counties. (Congressman Loudenslager, Woodbury, Rep.) Dr. Wm. B. E. Miller, Camden; Dr. John O. George, Camden; Dr. Charles E. Magill, Handonfield; Dr. Edgar H. Landes, Camden.

*Second Congressional District:* Atlantic, Mercer, Burlington, and Ocean Counties. (Congressman Gardner, Atlantic City, Rep.) Dr. A. T. Sellers, Atlantic City; Dr. L. P. Hurley,

Hopewell; Dr. G. F. Harker, Trenton: Dr. James M. Mecray, Maple Shade.

*Third Congressional District:* Somerset, Middlesex, and Monmouth Counties. (Congressman Howell, New Brunswick, Rep.) Dr. E. L. Loblein, New Brunswick; Dr. S. Lockwood, Woodbridge; Dr. B. F. King, Little Silver; Dr. E. R. Voorhees, Somerville: Dr. H. W. Read, Freehold.

*Fourth Congressional District:* Sussex, Warren, Hunterdon, and Mercer Counties. (Congressman Salmon, Boonton, Dem.) Dr. M. M. Stage, Dover; Dr. John M. Mitchell, Madison; Dr. G. P. Ellice, Morristown; Dr. H. W. Dustan, Morristown; Dr. A. H. McIntosh, Summit.

*Fifth Congressional District:* Passaic and Bergen Counties. (Congressman Stewart, Paterson, Rep.) Dr. S. S. Tredwell, Englewood; Dr. J. B. Finch, Ramsey; Dr. John Kehoe, Lyndhurst; Dr. R. O. Hasbrouck, Passaic; Dr. J. Payne Lowe, Passaic.

*Sixth Congressional District:* City of Newark and the township of East Orange, Essex County. (Congressman R. Wayne Parker, Newark, Rep.) Dr. T. Earle Budd, 90 Park St., Orange; Dr. Werner Runge, Newark; Dr. J. W. Hawk, Newark; Dr. Jas. T. Glennon, Newark; Dr. Andrew G. Vogt, Newark.

*Seventh Congressional District:* All of Hudson County excepting City of Bayonne. (Congressman Daly, Hoboken, Dem., deceased.) Dr. T. E. Smith, 309 Barrow St., Jersey City; Dr. Elisha Matthews, Jersey City; Dr. R. W. A. English, Jersey City.

*Eighth Congressional District:* The County of Union, the City of Bayonne (Hudson County), and all of the County of Essex excepting the City of Newark and the township of East Orange. (Congressman Fowler, Elizabeth, Rep.) Dr. F. A. Zucker, Elizabeth; Dr. M. O. M. Knott, Plainfield; Dr. W. F. Harrison, Bloomfield; Dr. James McDonough, Montclair; Dr. Raymond B. Smith, Montclair.

---

Anthrax has made its appearance in Eastern New Jersey among some very valuable herds of cattle. Dr. T. B. Rogers, Woodbury, has been placed in charge of the outbreak.

The new English journal on "Hygiene," which will appear about January 1, 1901, will have the assistance of Professor Leonard Pearson as one of the corps of collaborators.

